# The Structure and Measurement of Unusual Sensory Experiences in Different Modalities: The Multi-Modality Unusual Sensory Experiences Questionnaire (MUSEQ)

**DOI:** 10.3389/fpsyg.2017.01363

**Published:** 2017-08-11

**Authors:** Claire A. A. Mitchell, Murray T. Maybery, Suzanna N. Russell-Smith, Daniel Collerton, Gilles E. Gignac, Flavie Waters

**Affiliations:** ^1^School of Psychological Science, The University of Western Australia Crawley, WA, Australia; ^2^Northumberland, Tyne and Wear NHS Foundation Trust, Bensham Hospital Gateshead, United Kingdom; ^3^Institute of Neuroscience, Newcastle University Newcastle upon Tyne, United Kingdom; ^4^Clinical Research Centre, Graylands Hospital, North Metro Health Service Mental Health Mount Claremont, WA, Australia

**Keywords:** hallucination, voices, visions, sensed presence, perception, sensory modalities, self-report scale, proneness

## Abstract

Hallucinations and other unusual sensory experiences (USE) can occur in all modalities in the general population. Yet, the existing literature is dominated by investigations into auditory hallucinations (“voices”), while other modalities remain under-researched. Furthermore, there is a paucity of measures which can systematically assess different modalities, which limits our ability to detect individual and group differences across modalities. The current study explored such differences using a new scale, the Multi-Modality Unusual Sensory Experiences Questionnaire (MUSEQ). The MUSEQ is a 43-item self-report measure which assesses USE in six modalities: auditory, visual, olfactory, gustatory, bodily sensations, and sensed presence. Scale development and validation involved a total of 1,300 participants, which included: 513 students and community members for initial development, 32 individuals with schizophrenia spectrum disorder or bipolar disorder for validation, 659 students for factor replication, and 96 students for test-retest reliability. Confirmatory factor analyses showed that a correlated-factors model and bifactor model yielded acceptable model fit, while a unidimensional model fitted poorly. These findings were confirmed in the replication sample. Results showed contributions from a general common factor, as well as modality-specific factors. The latter accounted for less variance than the general factor, but could still detect theoretically meaningful group differences. The MUSEQ showed good reliability, construct validity, and could discriminate non-clinical and clinical groups. The MUSEQ offers a reliable means of measuring hallucinations and other USE in six different modalities.

## Introduction

The assessment and classification of psychopathology and other psychological constructs is historically categorical in nature. Some mental experiences, however, can occur on a continuum ranging from subthreshold levels in the general population to acute symptoms in clinical groups, and this observation has led to the exploration of symptom dimensions. In particular, the psychosis continuum theory (Johns and Van Os, 2001) suggests that the positive symptoms of psychosis (i.e., hallucinations and delusions) can exist at attenuated levels in the general population without being associated with distress or a loss of contact with reality that might warrant a need for treatment (Preti et al., 2014; Brett et al., 2015; Waters et al., 2017).

Although the psychosis continuum model is debated (Kaymaz and Van Os, 2010; Lawrie et al., 2010), there is overwhelming evidence that people in the general population report a range of hallucinations and other unusual sensory experiences (USE) (see Nuevo et al., 2010; Pechey and Halligan, 2012; McGrath et al., 2015). In this report, the term USE refers to a range of phenomena such as hallucinations and misperceptions where there is discrepancy between what is perceived and what actually exists in the real world. It is sometimes difficult to precisely distinguish hallucinations from other perceptual experiences given that perception is subjective and complex (Dror, 2005), and there is no clear-cut distinction between categories of experiences. Nonetheless, hallucinations are often defined as waking experiences which have the character of veridical perceptions, but are elicited in the absence of a relevant external stimulus. Misperceptions refer to experiences (images, sounds, etc.) whose relationship to stimuli in the outside world is distorted or changed in some way.

Studies of USE in the general population typically focus on hallucinations. Epidemiological studies estimate a lifetime prevalence of approximately 5–30% of (Linscott and Van Os, 2013; Kråkvik et al., 2015; McGrath et al., 2015; Peters et al., 2016). For about 80% of individuals who experience psychotic-like experiences (including hallucinations), these are transient experiences that tend to remit over time (Linscott and Van Os, 2013). In the remaining 20%, symptoms are persistent and distressing, and approximately 7% may go on to develop a psychotic disorder (Linscott and Van Os, 2013). Studies tend to screen community participants for psychiatric disorders, but it is possible that these experiences are related to prodromal psychosis, an undiagnosed medical condition (e.g., disease of the sensory organs), or other disorders (e.g., sleep disorders).

The auditory and visual modalities are the most commonly investigated, although investigators often tend to focus exclusively on one modality. The other sensory modalities however require further attention. USE can occur in different modalities (e.g., auditory, visual, olfactory, gustatory, and bodily sensations), as shown in a broad variety of clinical conditions (e.g., schizophrenia and other psychosis, neurodegenerative diseases, eye disease, and temporal lobe epilepsy) (see Lewandowski et al., 2009; Ford and Almeida, 2014; Larøi et al., 2014; Stephane et al., 2014; Waters et al., 2014a). Different modalities of USE have also been reported in the general population (Tien, 1991; Ohayon, 2000; Peters et al., 2016). Lifetime prevalence rates of auditory hallucinations range between 2.5 and 15% (e.g., Beavan et al., 2011; Kråkvik et al., 2015; McGrath et al., 2015). Similarly, visual experiences are relatively common with lifetime estimates ranging between 3 and 15%, and these prevalence rates escalate rapidly in association with increase age, visual loss, social isolation, and sleep deprivation (Tien, 1991; Ohayon, 2000; McGrath et al., 2015; Peters et al., 2016). Studies also suggest non-negligible lifetime estimates of olfactory hallucinations in approximately 3–9% of the population (Ohayon, 2000; García-Ptacek et al., 2013), gustatory hallucinations in 1% (Tien, 1991; Ohayon, 2000), and bodily/somatic hallucinations in 3–7% (Ohayon, 2000; Shevlin et al., 2007; Peters et al., 2016). Peters et al. (2016) even suggest that bodily and olfactory hallucinations may be more common in the general community than in psychosis. Sensed presence is another sensory domain which is underreported. Despite limited research, existing evidence suggests that the lifetime prevalence of sensed presence experiences may be as high as 30–60% in selected (e.g., bereaved) populations (Castelnovo et al., 2015).

Given the existence of USE in multiple sensory domains, comprehensive and sensitive assessment scales are required, although currently lacking. The development of a comprehensive and accurate measure of USE in different modalities is critical to furthering our understanding of these phenomena, and of their relation to other psychological constructs and mental health outcomes. Until now, the presence and frequency of hallucinations and other USE in the general population have been assessed rather broadly. For example, scales such as the Oxford-Liverpool Inventory of Feelings and Experiences (O-LIFE; Mason et al., 1995, 2005) which targets the schizotypy personality construct, assess unusual experiences via items relating to daydreaming, magical thinking, and paranoia. This limits the subscale's utility in examining specific perceptual changes.

The Launay Slade Hallucination Scale (LSHS; Launay and Slade, 1981) and its revisions (e.g., Revised Hallucination Scale; Morrison et al., 2000; LSHS-Modified; Larøi et al., 2004; LSHS-Modified-II [LSHS-M-II]; Larøi and Van der Linden, 2005), are the most commonly used measures of hallucination “proneness.” Recent versions of the LSHS (e.g., LSHS-M-II) target hallucinations in various modalities (auditory, visual, olfactory, tactile, and sensed presence) (Larøi and Van der Linden, 2005; Kråkvik et al., 2015). However, these modalities are not assessed in detail (i.e., one or two items per modality) and the scale was developed without theoretical underpinnings except to build on the notion of “proneness,” which does not have a clear definition or meaning. If the focus of research is on particular aspects of hallucinations in the community, the development of more internally consistent scale is required.

A more detailed focus on perceptual anomalies was achieved with the Cardiff Anomalous Perception Scale (CAPS; Bell et al., 2006). The CAPS assesses a range of perceptual anomalies in different modalities, but these are grouped into different phenomenological dimensions (e.g., changes in sensory intensity, sensory experience from an unexplained source, and thought echo) rather than by modality. Precise frequency labels are also lacking, which limits the assessment of frequency and persistence of symptoms which can signal a transition into a psychotic state (Stefanis et al., 2002; Dominguez et al., 2011). In summary, no measure is yet available to systematically explore the frequency of hallucination and USE in different modalities. The first aim of this study was therefore to create a scale that assesses USE in the six most common sensory modalities.

Scale development and validation, however, is not a simple task, particularly if different factors/modalities are thought to be separable. The ability to accurately measure USE in different modalities is dependent on the statistical separability of these experiences. Principal components analyses and exploratory methods were previously used to guide previous scale development (e.g., in the creation of the CAPS). These methods can be helpful for item reduction, although there is no *a priori* expectation regarding how items may be related.

One question to consider is whether USE are better explained by a single general “proneness” or “hallucination” factor, or whether the different modalities are sufficiently unique to lead to individual differences. Previous findings have indicated significant correlations between hallucinations in different modalities within the same individuals (e.g., Launay and Slade, 1981; Morrison et al., 2000; Larøi and Van der Linden, 2005; Bell et al., 2006; Lewandowski et al., 2009; Preti et al., 2014). However, evidence from phenomenological and neurobiological studies also indicated that USE can be differentiated at the modality level (Weiss and Heckers, 1999; Larøi, 2006; Jardri et al., 2013; Waters et al., 2014b), such that individuals may only ever experience hallucinations in one modality. Since both approaches appear valid, we put forward a third possiblity which incorporates both views, and which proposes one large general factor as well as unique variance from different modalities.

This hybrid approach has support from other domains of psychology. For example, Lahey et al. (2012) proposed that high correlations between dimensions of general psychopathology (externalizing, internalizing, and thought disorder) may be reflective of a general factor representing an individual's predisposition to developing psychopathology, but with additional separate factors which act to shape this vulnerability into a specific symptom manifestation. Statistical methods which employ confirmatory factor analysis using a bifactor modeling approach (e.g., Caspi et al., 2014; Laceulle et al., 2015; Waldman et al., 2016) also support this model.

A bifactor modeling approach assessing for the presence of a general factor and (typically correlated) domain-specific latent factors has also been used successfully in psychosis research by Reininghaus et al. (2013). Their findings demonstrated the presence of a general psychosis factor as well as individual symptom dimensions. This has been replicated in a general population sample (Shevlin et al., 2016). Interpretations of what the general factor represents include a general risk or common etiology, associated distress or impairment, or a level of symptom acuity (Lahey et al., 2012; Caspi et al., 2014; Waldman et al., 2016).

This hierarchical structure is yet to be explored in relation to different sensory modalities of USE, and to do so is of great importance in furthering our understanding of the commonalities and separability of these phenomena. It also allows us to explore conceptual issues relating modality-specific vs. generalized deficits. Indeed, the nature of hallucinations in individuals with psychosis, in which the presence of hallucinations in the auditory and visual modalities increases the chance of additional hallucinations (e.g., tactile and olfactory) (see Lim et al., 2016; Clark et al., 2017), suggests related but potentially differentiable “vulnerability” mechanisms.

Notwithstanding the possibility of a general USE factor, evidence exists of independent sensory contributions. Examples include schizophrenia spectrum disorders, which are characterized predominantly by auditory hallucinations in contrast to other medical conditions and psychiatric disorders, in which visual hallucinations are more common (Lewandowski et al., 2009; Waters et al., 2014a). Another example includes an overrepresentation of visual hallucinations in developing countries compared to Western cultures (Luhrmann, 2011). Yet another example is that of the role of childhood trauma mpacting on the specific modality of USE, with tactile hallucinations being linked to physical abuse and visual hallucinations with neglect (Shevlin et al., 2007). Factors which serve to accentuate the expression of one modality over another therefore appear to include neurobiological mechanisms, culture, and psychosocial factors.

Such findings indicate that USE should be characterized by multiple sensory dimensions, and also indicate the importance of assessing both a general factor and individual modalities. Confirmatory approaches are preferred in scale development where theoretical knowledge anticipates a relationship between items (Ten Holt et al., 2010). Exploratory approaches, by contrast, do not allow for the testing of multidimensionality or higher-order constructs which may result in an inappropriate solution (McGartland Rubio et al., 2001). The second aim of this study was therefore to perform a series of confirmatory factor analyses to assess the factor structure of the new scale. Specifically, we aimed to test alternative models of the factorial structure of USE as a way of exploring their potential separability at the modality level.

In summary, existing measures of USE have contributed to a rich body of knowledge but are limited in their ability to systematically assess different modalities. Approaches toward scale development also need to be more systematic and theoretically guided to validly measure USE in their respective modalities. Specifically, the possibility of a model structure comprising a general factor and modality-specific factors is yet to be explored regarding USE. Therefore, the aims of the current study were to:

1. Create a scale that separately assesses USE in six common sensory modalities, and
2. Evaluate the psychometric properties of this scale, while testing alternative factor structure models to explore the contribution of general and specific factors.

We report on the development and psychometric properties of the resulting scale, called the Multi-Modality Unusual Sensory Experiences Questionnaire (MUSEQ). The development of the MUSEQ was theory-driven with *a priori* hypotheses regarding the relationship between modalities. Specifically, it was hypothesized that all items (and modalities) would be positively related to each other, but still be separable into modality-specific domains. Results regarding the factorial structure are checked in a replication sample.

## Method

### Participants

#### Non-clinical

Six-hundred and sixteen participants took part in the initial scale development study. Forty participants were excluded due to incomplete questionnaires. Recruitment targeted students (*N* = 298) and non-student general community volunteers (*N* = 215) aged 17 and above. Students were recruited via an undergraduate student pool at the University of Western Australia and through research and social media websites (Call for Participants, Psychological Research on the Net, Online Psychology Research UK, The Inquisitive Mind, PsyResearch, Social Psychology Network, Intervoice, U3A Online, Facebook, Find Participants, and CrowdFlower). The community sample was also obtained via these websites. To ensure endorsed USE were not due to other psychiatric and neurological conditions, exclusion criteria included a self-reported history of diagnosed schizophrenia spectrum disorder or bipolar disorder (*N* = 31), epilepsy (*N* = 10), neurodegenerative disease (*N* = 4), and traumatic brain injury associated with loss of consciousness (*N* = 18).

The final sample comprised 513 participants (386 female, 127 male), with a mean age of 27.75 years (*SD* = 13.28, range 17–76; *N* = 489). Participants were drawn from Australia (48.7%), United States of America/Canada (42.8%), United Kingdom (5.3%), and other countries (3.2%), with most obtaining an education level of high school or greater (High School 53.4%; Tertiary Studies 37.0%; other 9.6%). Participants who were not students (*N* = 215) predominantly worked full-time (50.2%), followed by part-time work (20%), unemployment (14%), retirement (8.4%), casual work (5.6%), and volunteering (1.9%). Reported marital status was as follows: Single (69.4%), Married (14.8%), De Facto (9.9%), Divorced (4.3%), and Widowed (1.6%). Religious affiliations were: Atheism (44.8%), Christianity (39.4%), Buddhism (2.9%), Islam (2.3%), Judaism (1.2%), Hinduism (1.0%), other (6.2%), and undisclosed (2.1%).

#### Clinical

Thirty-two individuals (20 female, 12 male) with schizophrenia spectrum disorder (*N* = 10) or bipolar disorder (*N* = 14) completed the MUSEQ (age *M* = 34.17 years, *SD* = 13.09, range 18–67) for validation purposes. Participants reported their diagnosis was made by a medical or mental health practitioner. A subset of eight participants chose not to disclose which of these two diagnostic categories pertained to them. Recruitment occurred via outpatient hospital clinics (*N* = 6) and online research websites (as above).

#### Replication and test-retest samples

Two additional samples were recruited via the undergraduate student pool at the University of Western Australia. Applying the same exclusion criteria as used for the original sample, 659 students (438 female, 221 male, *M* age = 20.92 years, *SD* = 5.58, range 16–71) were used to test the replicability of the factor structure. A further 96 students (68 female, 27 male, 1 “other”; *M* age = 19.82 years, *SD* = 4.49, range 17–41) completed the MUSEQ at two time points (6 months apart) to determine test-retest reliability.

### Questionnaires

#### Demographic and health questionnaire

The non-clinical sample (*N* = 513) completed 13 questions relating to demographic information (e.g., age, gender, education level, and marital status). There were also 10 questions covering lifetime medical history (sleep disorder, epilepsy, traumatic head injury, neurodegenerative disease), mental health history (including schizophrenia spectrum disorder and bipolar disorder), and sensory-related conditions or experiences (eye problems/disease not including glasses/contact prescriptions, ear/nose/throat problems, and synaesthesia). The year of diagnosis was requested for any questions positively endorsed (where applicable).

#### Cardiff anomalous perception scale (CAPS)

The CAPS (Bell et al., 2006) is a 32-item self-report measure that assesses a range of perceptual anomalies (e.g., sensory intensity, sensory flooding, and hallucinations) on a dichotomous “Yes” or “No” scale. If items are positively endorsed, participants are required to rate the distress (“Not at all distressing” to “Very distressing”), intrusiveness (“Not at all distracting” to “Completely intrusive”), and frequency (“Happens hardly at all” to “Happens all the time”) associated with that experience on a 1–5 Likert scale. Total scores range from 0 to 32 (for the dichotomous responses) and subscale scores range from 0 to 160 (for the five-point responses).

#### Launay slade hallucination scale-modified-II (LSHS-M-II)

The LSHS-M-II (Larøi and Van der Linden, 2005) is a 16-item self-report scale assessing hallucination proneness. This study used Larøi and Van der Linden's (2005) adaptation of the scale, but excluded the follow-up questions. Items are rated on a five-point Likert scale (0: “*Certainly does not apply to me*” to 4: “*Certainly applies to me*”). Potential scores range from 0 to 60.

#### Oxford-liverpool inventory of feelings and experiences—short version (O-LIFE-S)

The O-LIFE-S (Mason et al., 2005) is a 43-item self-report measure of schizotypal traits. We utilized three subscales: unusual experiences (UE; 12 items), cognitive disorganization (CD; 11 items), and introvertive anhedonia (IA; 10 items). Items are rated on a dichotomous “Yes” or “No” scale, and potential scores range from zero to the total number of items in each subscale.

### Scale development

#### Item creation

The objective of the item creation stage was to identify a range of questions to assess USE according to a continuum structure. This included items ranging from the most frequent phenomena (likely to be strongly endorsed) through to “borderland”-like perceptual experiences, which are experienced as external and non-self (i.e., akin to clinical phenomena). This continuum was informed by theoretical work which describes the qualitative ranges in consciousness and subjectivity that occur in schizotypy, the prodrome phase of psychosis, and in some organic, medical, or affective states (Meehl, 1962; Jansson, 2014).

An extensive review was conducted of scales and interviews that assess hallucination proneness, delusional ideation, psychosis proneness, schizotypy, perceptual anomalies, and hallucinations in different clinical groups (see Table 1). Items were selected in six modalities (auditory, visual, olfactory, gustatory, bodily sensations, and sensed presence). This review resulted in a list of 82 items, which were used as a foundation to create the new scale items.

**Table 1:** Scales and assessment measures reviewed during development of the MUSEQ.

**Self-report scales**		**Clinical interview schedules/Clinician rated assessment**	
Cardiff Anomalous Perception Scale Bell et al., 2006 Community Assessment of Psychic Experiences Stefanis et al., 2002 Launay Slade Hallucination Scale Launay and Slade, 1981 Revised Hallucination Scale Morrison et al., 2000 Launay Slade Hallucination Scale-Modified Larøi et al., 2004 Launay Slade Hallucination Scale-Modified-II Larøi and Van der Linden, 2005 Magical Ideation Scale Eckbald and Chapman, 1983 Oxford-Liverpool Inventory of Feelings and Experiences Mason et al., 1995, 2005 Perceptual Abberation Scale Chapman et al., 1978	Peters et al. Delusions Inventory Peters et al., 1999 Schizotypal Personality Questionnaire-Brief Raine and Benishay, 1995 Self-Rated Visual Hallucination Questionnaire for Parkinson's Disease Barnes and David, 2001	Columbia University Scale for Psychopathology in Alzheimer's Disease Devanand et al., 1992 Examination of Anomalous Self-Experience Parnas et al., 2005 Geriatric Mental State Schedule Copeland et al., 1976 Institute of Psychiatry Visual Hallucinations Interview Santhouse et al., 2000 Mental Health Research Institute Unusual Perceptions Schedule Copolov and Carter, 1995 Neuropsychiatric Inventory Cummings et al., 1994 North-East Visual Hallucinations Interview Mosimann et al., 2008 Olfactory Hallucinations Phenomenological Survey Stevenson et al., 2011 Parkinson's Psychosis Questionnaire Brandstaedter et al., 2005) Present State Examination Wing et al., 1974 Psychotic Symptom Rating Scales Haddock et al., 1999	Queens Square Visual Hallucination Inventory Williams et al., 2008 Scale for Olfactory Hallucinations Kwapil et al., 1996 Scale for the Assessment of Positive Symptoms Andreasen, 1984 Scale for the Assessment of Positive Symptoms of Parkinson's Disease Voss et al., 2013 Schedules for Clinical Assessment in Neuropsychiatry Wing et al., 1990 Semi-Structured Interview About Visions in Psychiatric Patients Gauntlett-Gilbert and Kuipers, 2005 Semi-Structured Interview on Complex Visual Hallucinations for Charles Bonnet Syndrome Teunisse et al., 1996 Structured Interview for Assessing Perceptual Anomalies Bunney et al., 1999 University of Miami Parkinson's Disease Hallucination Questionnaire Papapetropoulos et al., 2008

With the exception of sensed presence, items in each modality were created to encompass a range of experiences corresponding with the following general continuum structure: item 1 = broad subclinical sensory experiences (e.g., senses playing tricks), item 2 = changes in perceptual intensity, item 3 = internal events become externalized, item 4 = misperceptions, and item 5 (and above) = hallucinations (having a non-self origin). Consultation was sought with an expert in the field throughout the scale development process to confirm item suitability.

#### Item selection

A brief exploratory factor analysis was initially conducted on each subscale separately (one-factor) to aid with item selection analysis. Following Osborne and Costello's (2009) guidelines, item analysis involved checking items had appropriate communalities (>0.40) and strong factor loadings (>0.50) within their respective subscale. Only one bodily sensation item, which related to feeling vibration on one's body with no apparent stimulus, was removed due to a low communality and weak factor loading. All other items had adequate communalities, and medium to strong (0.40–0.79) factor loadings within their respective subscales. The items were piloted with participants from the general population (*N* = 8) who provided feedback on their relevance, phrasing, and comprehensibility.

The final scale comprised 43 questions organized across six modality subscales: auditory (7 items), visual (8 items), olfactory (8 items), gustatory (8 items), bodily sensations (8 items), and sensed presence (4 items) (see Table 2). Items are rated on a five-point Likert scale that targets the frequency of USE, specifically adapted for a non-clinical population (0 = *Never* [Never Happened]; 1 = *Hardly Ever* [Once or twice in my life]; 2 = *Rarely* [Once or twice a year]; 3 = *Occasionally* [A few times a year]; 4 = *Frequently* [At least monthly]. The MUSEQ is available online (see Supplementary Table 4)

**Table 2:** Final MUSEQ items.

**There have been times when**...
**AUDITORY**
A1. My ears have played tricks on me A2. Sounds were louder than they normally would be A3. I thought of a song and could almost hear it with distinct clarity A4. I was in a crowd or with other people and heard my name being called, only to find that I was mistaken A5. I have heard my phone ring then found that it wasn't ringing at all A6. I could hear sounds, music, or noises that other people could not hear A7. I have heard a person's voice and then found that no-one was there
**VISUAL**
V1. My eyes have played tricks on me V2. I found that lights or colors seem brighter or more intense than they normally would be V3. I thought of people, objects, or landscapes, and could almost see their image in front of my eyes V4. I have looked at a patterned object (e.g., wallpaper, curtains, tiled floor) and a figure or face has emerged V5. I have seen lights, flashes, or other shapes that other people could not see V6. I looked at an object and it transformed itself before my eyes into something else V7. I saw a brief image of an object, animal, or person pass me by in my peripheral vision, but when I looked there was nothing there V8. I saw people, faces, or animals, and then found that nothing was there
**OLFACTORY**
O1. My nose (sense of smell) has played tricks on me O2. I thought that everyday smells were unusually strong O3. I thought of a smell and I could almost smell it for real O4. Common smells seemed unusually different O5. I noticed the smell of smoke, burning, or gas when there was nothing there O6. I have suddenly been struck by an unpleasant or disgusting smell that no-one else could smell O7. I have suddenly been struck by a very pleasant smell that no-one else could smell O8. I have been struck with the smell of odd things which I interpreted as death, colors, or ghosts
**GUSTATORY**
G1. My sense of taste has played tricks on me G2. I thought that food or drink tasted stronger than it normally would G3. I thought of a taste and found that I could taste it in my mouth as if it was real G4. I ate the same food as another person and thought it tasted off, but the other person did not seem to think so G5. I have consumed food or drink and it tasted like something completely different G6. I had nothing in my mouth but I suddenly tasted something very confusing which faded very quickly G7. I had nothing in my mouth but I suddenly tasted something unpleasant which was really persistent G8. I had nothing in my mouth but I suddenly tasted something very pleasant which was really persistent
**BODILY SENSATIONS**
BS1. My body senses have played tricks on me BS2. I found my skin to be more sensitive to cold, heat, or touch than usual BS3. I thought of a touch or other sensations on my skin and almost felt it on my skin BS4. I have experienced the sensation that my body (or part of my body) was different in shape or size BS5. I could feel burning, tingling, scraping, or heat on my skin, although there was nothing causing it BS6. I have felt things moving or crawling on or under my skin BS7. I have experienced the sensation that something was pressing on my skin, or that I was holding an object in my hand, but then found there was nothing there BS8. I have felt someone or something touching me, but when I turned to look there was nothing there
**SENSED PRESENCE**
SP1. I felt the presence of someone, even though I could not see them (e.g., behind me, or in another room) SP2. I have felt an unseen evil presence around me SP3. I have felt an unseen angelic presence around me SP4. I have felt the presence of a relative or friend who has passed away

### Procedure

Participants anonymously completed the self-report questionnaires on Qualtrics software accessed via a secure internet hyperlink, or via hard copy administration. The non-clinical sample (*N* = 513) completed the MUSEQ, O-LIFE-S, LSHS-M-II, and CAPS, whereas the clinical sample (*N* = 32), replication sample (*N* = 659), and test-retest sample (*N* = 96) completed the MUSEQ only. This study and its protocols were approved by the Human Research Ethics Committee of the University of Western Australia. All participants provided written informed consent prior to their participation.

### Statistical analyses

**Figure 1:**
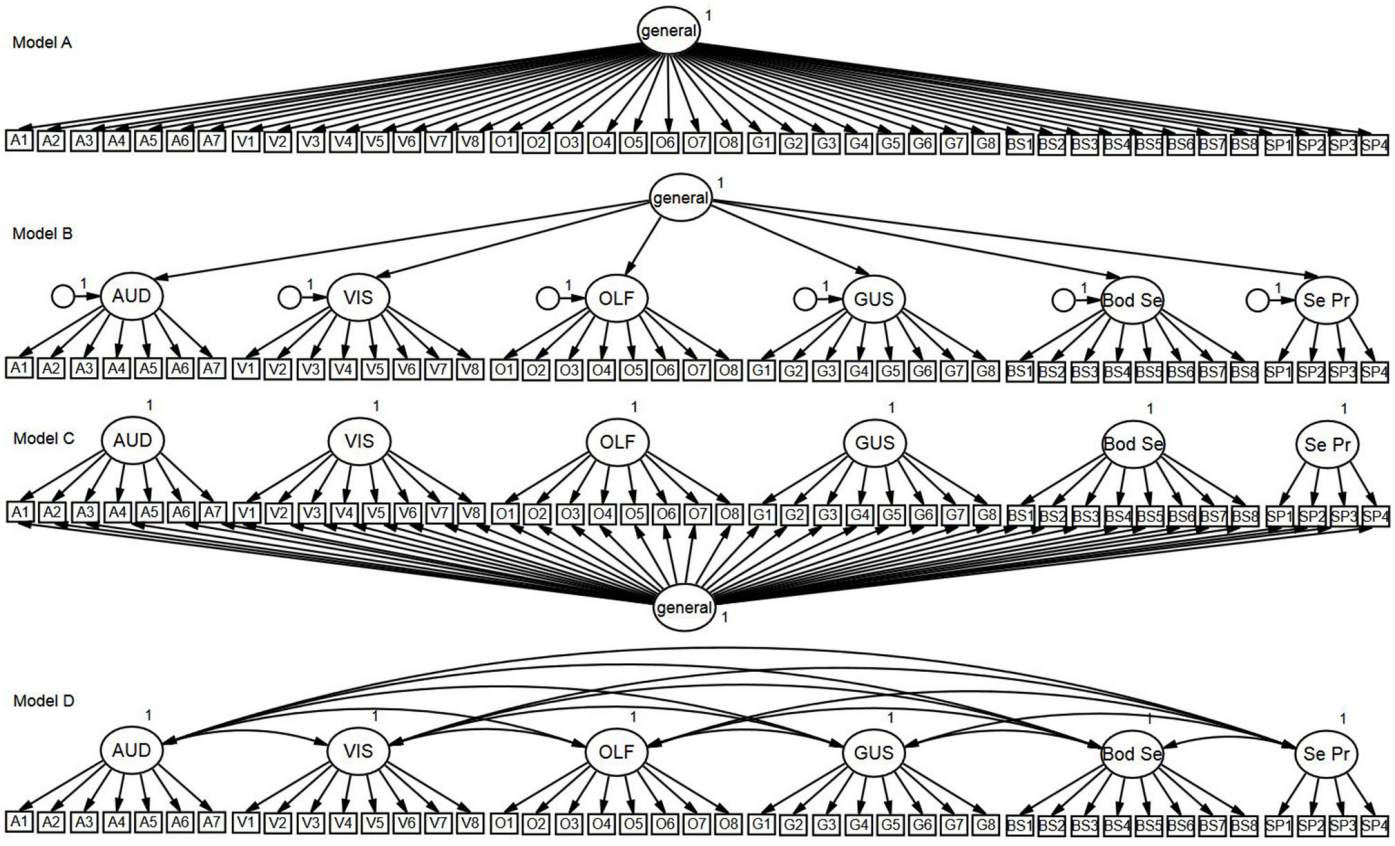
Confirmatory factor analysis models. Model A, One-Factor Model; Model B, Second-Order Model; Model C, Bifactor Model; Model D, Correlated-Factors Model.

A series of confirmatory factor analyses were performed using MPlus 7 (Muthén and Muthén, 1998-2012) in the original sample (*N* = 513) in order to assess the factor structure of the new scale. Due to the non-normal and ordinal nature of the data, analyses were based on polychoric correlations, and a weighted least squares means and variance adjusted (WLSMV) estimation was used (Brown, 2015). For the purposes of factor identification, all latent variable variances in the model were constrained to 1, except the second-order model. The second-order model first-order factors were scaled by constraining a loading from each to 1. Model comparisons were facilitated by testing a series of four progressively more complex models that have been frequently used in studies examining the structure of hierarchical constructs (e.g., Reininghaus et al., 2013; Caspi et al., 2014; Laceulle et al., 2015):

- One-factor model: Tested the hypothesis that all items are influenced by a general factor and are unidimensional (see Figure 1 Model A).
- Second-order (higher-order) model: Tested the hypothesis that the covariance between the modality-specific latent factors is explained by a higher-order general latent factor. In this model, there is no direct relationship between the general factor and individual items; rather, the relationship is indirectly mediated by the modality-specific latent factors (see Figure 1 Model B).
- Bifactor model: Tested the hypothesis that all items load onto both a general factor and one of the six modality-specific factors (see Figure 1 Model C). For example, all auditory items load onto the general factor and the auditory latent factor, but no other modality-specific factor. This model specifies a general factor that may reflect what is common between the items, while also being able to simultaneously test the unique variance associated with each modality factor. Thus, the model allows both the general factor and specific modality factors to have direct influence on the items, but unlike the second-order model, the specific modality factors do not mediate the influence of the general factor.
- Correlated-factors (six-factor) model: Tested the hypothesis that each modality forms a latent factor, with each factor influencing a subset of USE in the respective modality (see Figure 1 Model D). This model assumes that the latent modality factors may be correlated.

These four models were re-tested in the replication sample.

Acceptable model fit was determined using the following criteria: a relative chi-square statistic <3, root mean square error of approximation (RMSEA) values <0.08 (good absolute close-fit <0.05), and comparative fit index (CFI) and Tucker-Lewis Index (TLI) values >0.90 (good incremental close-fit >0.95) (Schweizer, 2010). Model comparisons were based on the degree of practical improvement in the models, as determined by a TLI difference of 0.10 or greater (Gignac, 2016). The use of a bifactor model also provided the opportunity to calculate omega subscale (ω_s_) and omega hierarchical (ω_h_) coefficients. The ω_s_ measures the degree to which the subscales are reliable and interpretable after controlling for the general factor, while the (ω_h_) represents the degree to which the general factor can be interpreted as reflective of a single common construct while controlling for the modality factors (Zinbarg et al., 2005; Reise et al., 2013). There are currently no specific guidelines for interpreting ω_s_ and ω_h_ coefficients, but Gignac and Kretzschmar (2017) proposed relative values of <0.20 (relatively small), 0.20–0.30 (typical), and >0.30 (relatively large) for ω_s_ based on a quantitative survey of the published literature.

Internal consistency reliability was estimated for each of the six modality subscales and calculated using the Cronbach's α coefficient. Test-retest reliability was determined using a Pearson's correlation between MUSEQ scores at two time points (6 months apart). Convergent and discriminant validity was assessed by correlating the MUSEQ total and subscale scores with the LSHS-M-II, CAPS, and O-LIFE-S subscales using Pearson's *r*. The ability of the scale to discriminate between non-clinical and clinical groups was measured by comparing mean differences (*t-*tests) on the total and subscale MUSEQ scores between the non-clinical sample and clinical sample. Incremental validity was assessed using *t*-tests on the data of participants with and without a history of sleep disorder, eye problems/disease, and synaesthesia. These three variables were selected due to sufficient sample size compared to other health variables recorded during the study. These comparisons allow us to demonstrate the usefulness of assessing modalities separately, rather than measuring USE as a unidimensional construct (i.e., total score). Given the non-normality of the data, correlation and mean difference analyses used bootstrapping (1,000 samples) to estimate the *p*-values and 95% confidence intervals. Descriptive and inferential analyses were performed using SPSS Statistics Version 22.

## Results

### Factor structure

#### One-factor model: is a unidimensional model suitable?

As can be seen in Table 3, the one-factor model (Figure 1 Model A) was associated with unacceptable levels of absolute (RMSEA) and incremental close-fit (CFI and TLI). Factor loadings are shown in Table 4. These results indicated that a unidimensional general factor was not appropriate in explaining the relationship between the MUSEQ items.

**Table 3:** Model fit statistics associated with the confirmatory factor analysis models for the original and replication samples.

	**Original sample (*N* = 513)**	**Replication sample (*N* = 659)**
**MODEL STATISTICS**		
**Null (Baseline)**		
χ^2^	21,842.31	32,253.55
*df*	903	903
χ^2^:*df*	24.19	35.72
RMSEA [90% CI]	0.213 [0.194, 0.231]	0.230 [0.210, 0.249]
CFI	0.000	0.000
TLI	0.000	0.000
**A: One-Factor**		
χ^2^	3,851.21	4,632.97
*df*	860	860
χ^2^:*df*	4.48	5.39
RMSEA [90% CI]	0.082 [0.080, 0.085]	0.082 [0.079, 0.084]
CFI	0.857	0.880
TLI	0.850	0.874
**B: Second-Order**		
χ^2^	2,136.61	2,834.77
*df*	854	854
χ^2^:*df*	2.50	3.32
RMSEA [90% CI]	0.054 [0.051, 0.057]	0.059 [0.057, 0.062]
CFI	0.939	0.937
TLI	0.935	0.933
**C: Bifactor**		
χ^2^	1,978.25	2,621.57
*df*	817	817
χ^2^:*df*	2.42	3.21
RMSEA [90% CI]	0.053 [0.050, 0.056]	0.058 [0.055, 0.060]
CFI	0.945	0.942
TLI	0.939	0.936
**D: Correlated-Factors**		
χ^2^	1,978.82	2,468.66
*df*	845	845
χ^2^:*df*	2.34	2.92
RMSEA [90% CI]	0.051 [0.048, 0.054]	0.054 [0.052, 0.056]
CFI	0.946	0.948
TLI	0.942	0.945

*χ^2^, chi-square; χ^2^:df, degrees of freedom, relative chi-square; RMSEA, root mean square error of approximation; CI, confidence interval; CFI, Comparative Fit Index; TLI, Tucker-Lewis Index*.

**Table 4:** Standardized factor loadings (WLSMV) for the one-factor and second-order models in the original sample (*N* = 513).

	**Model A (one-factor)**	**Model B (second-order)**						
**Item**	**General**	**A**	**V**	**O**	**G**	**BS**	**SP**	**General**
***A***								0.84
A1	00.63	0.73						
A2	0.61	0.71						
A3	0.52	0.61						
A4	0.50	0.58						
A5	0.50	0.59						
A6	0.65	0.76						
A7	0.68	0.79						
***V***								0.91
V1	0.72		0.79					
V2	0.67		0.73					
V3	0.65		0.70					
V4	0.62		0.67					
V5	0.72		0.79					
V6	0.71		0.77					
V7	0.73		0.79					
V8	0.76		0.82					
***O***								0.85
O1	0.67			0.76				
O2	0.66			0.74				
O3	0.63			0.71				
O4	0.73			0.82				
O5	0.64			0.72				
O6	0.70			0.78				
O7	0.74			0.82				
O8	0.73			0.82				
***G***								0.83
G1	0.73				0.82			
G2	0.70				0.79			
G3	0.67				0.76			
G4	0.66				0.75			
G5	0.72				0.80			
G6	0.74				0.83			
G7	0.73				0.81			
G8	0.79				0.87			
***BS***								0.89
BS1	0.75					0.82		
BS2	0.68					0.75		
BS3	0.70					0.77		
BS4	0.62					0.68		
BS5	0.65					0.71		
BS6	0.58					0.64		
BS7	0.76					0.83		
BS8	0.77					0.84		
***SP***								0.70
SP1	0.62						0.85	
SP2	0.60						0.81	
SP3	0.59						0.80	
SP4	0.48						0.65	

*Factor loadings in bold not significant (p > 0.05). A, Auditory; V, Visual; O, Olfactory; G, Gustatory; BS, Bodily Sensations; SP, Sensed Presence*.

#### Second-order model: is the covariance between modalities explained by a higher-order general factor?

The second-order model (Figure 1 Model B) yielded acceptable absolute and incremental close-fit. All standardized loadings were significant (*p* < 0.001) and positive (see Table 4). Loadings between the general factor and the modality factors ranged from 0.70–0.91 (all *p* < 0.001). The TLI difference >0.10 in favor of Model B vs. Model A suggested practical improvement in model fit for Model B (compared to Model A) (see Table 3).

#### Bifactor model: can the variance be explained by both a general factor and modality-specific factor?

The bifactor model (Figure 1 Model C) yielded acceptable absolute and incremental close-fit (see Table 3). All items loaded significantly (*p* < 0.001, item V3 *p* = 0.01) onto their respective modality factors, with the exception of item V2 on the visual factor (see Table 5). All items loaded significantly (*p* < 0.001) on the general factor, ranging from 0.45 to 0.77. There was practical improvement in model fit (TLI difference >0.10) from Model A to C, but not from Model B to C (see Table 3).

**Table 5:** Standardized factor loadings (WLSMV) for the bifactor and correlated-factors models in the original sample (*N* = 513).

		**Model C (bifactor)**						**Model D (correlated-factors)**					
**Item**	**General**	**A**	**V**	**O**	**G**	**BS**	**SP**	**A**	**V**	**O**	**G**	**BS**	**SP**
***A***									0.81	0.68	0.66	0.75	0.61
A1	0.61	0.39						0.73					
A2	0.61	0.26						0.71					
A3	0.53	0.19						0.61					
A4	0.46	0.51						0.58					
A5	0.48	0.43						0.59					
A6	0.64	0.42						0.76					
A7	0.67	0.40						0.79					
***V***										0.73	0.70	0.83	0.70
V1	0.72		0.26						0.78				
V2	0.69		**0.09**						0.73				
V3	0.66		0.12						0.70				
V4	0.61		0.32						0.67				
V5	0.72		0.26						0.79				
V6	0.69		0.37						0.77				
V7	0.70		0.47						0.79				
V8	0.73		0.49						0.82				
***O***											0.80	0.73	0.57
O1	0.65			0.39						0.76			
O2	0.62			0.49						0.74			
O3	0.62			0.23						0.71			
O4	0.71			0.35						0.82			
O5	0.62			0.34						0.72			
O6	0.63			0.58						0.78			
O7	0.68			0.54						0.82			
O8	0.74			0.16						0.82			
***G***												0.73	0.49
G1	0.66				0.52						0.81		
G2	0.61				0.62						0.79		
G3	0.67				0.22						0.76		
G4	0.63				0.37						0.75		
G5	0.66				0.47						0.80		
G6	0.69				0.43						0.83		
G7	0.67				0.44						0.80		
G8	0.73				0.45						0.87		
***BS***													0.62
BS1	0.73					0.33						0.82	
BS2	0.66					0.36						0.75	
BS3	0.68					0.38						0.77	
BS4	0.61					0.30						0.68	
BS5	0.61					0.48						0.71	
BS6	0.54					0.41						0.64	
BS7	0.73					0.39						0.83	
BS8	0.77					0.24						0.84	
***SP***													
SP1	0.61						0.39						0.85
SP2	0.56						0.63						0.81
SP3	0.54						0.68						0.80
SP4	0.45						0.51						0.65

*Factor loadings in bold not significant (p > 0.05). A, Auditory; V, Visual; O, Olfactory; G, Gustatory; BS, Bodily Sensations; SP, Sensed Presence*.

In order to determine the strength and importance of the modality factors in the bifactor model, the ω_s_ coefficients and ω_h_ coefficient were estimated. Modality subscales were associated with the following ω_s_ estimates: auditory ω_s_ = 0.26 (typical); visual ω_s_ = 0.14 (relatively small); olfactory ω_s_ = 0.24 (typical); gustatory ω_s_ = 0.29 (typical); bodily sensations ω_s_ = 0.21 (typical); and sensed presence ω_s_ = 0.44 (relatively large). The general factor was found to be associated with a ω_h_ = 0.92. Thus, the subscales appear to account for a relatively small amount of unique variance compared to the variance explained by the general factor.

#### The correlated-factors model: six modality-specific dimensions

As shown in Table 3, the correlated-factors model (Figure 1 Model D) fit the data well and was associated with acceptable levels of absolute and incremental close-fit. Standardized factor loadings (see Table 5) were all positive and significant at the *p* < 0.001 level. The correlations between the sensory modality factors ranged from 0.49 to 0.83, with the strongest correlations yielded between the visual and bodily sensations factors (*r* = 0.83), followed by the auditory and visual factors (*r* = 0.81). The TLI difference >0.10 in favor of Model D from Model B suggested practical improvement in model fit for Model D compared to Model B (see Table 3).

Direct comparisons between Model C (bifactor) and Model D (correlated-factors) were not possible as they are not nested models, and the WLSMV estimator does not provide estimations of alternative comparative fit indices (e.g., Akaike information criterion or Bayesian information criterion). Both models appeared to fit the data relatively well, with Model D being the most parsimonious model. A comparison of the specific modality factor loadings for Model C and Model D (see Table 5) found that, generally, loadings were greatly reduced when the general factor was removed in the bifactor model (e.g., item V1 reduced from 0.78 to 0.26). This suggested that the general factor accounted for a majority of the variance between USE in different modalities. However, it is important to note that for some items the difference was minimal (e.g., item A4 Model C = 0.51, Model D = 0.58 and item SP3 Model C = 0.68, Model D = 0.80), which suggests that the specific modality factors still account for some unique variance.

### Descriptive statistics

Given the findings of the factor analyses, the 43-item scale with six modality factors was conceptualized as the Multi-Modality Unusual Sensory Experiences Questionnaire (MUSEQ). Summing item responses on the 0–4 Likert scale, total scores can range from 0 to 172, with possible subscale scores as follows: auditory (0–28), visual (0–32), olfactory (0–32), gustatory (0–32), bodily sensations (0–32), and sensed presence (0–16). Table 6 presents the descriptive statistics associated with the MUSEQ for the non-clinical and clinical samples, as well as the LSHS-M-II, CAPS, and O-LIFE-S for the non-clinical sample.

**Table 6:** Non-clinical and clinical descriptive statistics for the MUSEQ and validation questionnaires.

**Scale**	**MUSEQ**							**LSHS-M-II (*N* = 500)**	**CAPS (*****N*** = **498)**				**O-LIFE-S (*****N*** = **503)**		
	**Total**	**A**	**V**	**O**	**G**	**BS**	**SP**	**Total**	**Total**	**D**	**I**	**F**	**UE**	**CD**	**IA**
**NON-CLINICAL (*****N*** = **513)**															
*M (SD)*	50.50 (29.94)	12.35 (6.14)	10.00 (7.21)	7.03 (6.17)	6.64 (5.97)	10.61 (7.52)	3.87 (3.44)	21.48 (12.61)	5.51 (5.31)	12.54 (14.71)	13.59 (15.77)	11.69 (13.86)	3.76 (2.74)	4.95 (3.18)	2.73 (2.17)
Median	47	12	9	6	5	10	3	20.50	4	8	8	7	3	5	2
Mode	28	14	5	0	0	0	0	13	0	0	0	0	2	3	2
Min - Max	0–151	0–28	0–32	0–30	0–31	0–32	0–16	0–56	0–31	0–108	0–108	0–98	0–12	0–11	0–9
**CLINICAL (*****N*** = **32)**															
*M* (*SD*)	79.38 (34.28)	18.63 (5.90)	15.91 (7.99)	11.16 (8.71)	9.63 (7.80)	16.28 (9.17)	7.78 (4.34)	–	–	–	–	–	–	–	–
Median	80	19	17	9	8	17	8	–	–	–	–	–	–	–	–
Mode	80	25	10	4	8	17	8	–	–	–	–	–	–	–	–
Min - Max	15–143	5–28	3–32	0–30	0–26	0–32	0–16	–	–	–	–	–	–	–	–

*MUSEQ, Multi-Modality Unusual Sensory Experiences Questionnaire; A, Auditory; V, Visual; O, Olfactory; G, Gustatory; BS, Bodily Sensations; SP, Sensed Presence; LSHS-M-II, Launay Slade Hallucination Scale-Modified-II; CAPS, Cardiff Anomalous Perception Scale; D, Distress; I, Intrusiveness; F, Frequency; O-LIFE-S, Oxford-Liverpool Inventory of Feelings and Experiences-Short Version; UE, Unusual Experiences; CD, Cognitive Disorganization; IA, Introvertive Anhedonia*.

### Reliability

#### Internal consistency

The MUSEQ subscales possess good internal consistency with Cronbach's alpha coefficients as follows: auditory α = 0.82; visual α = 0.88; olfactory α = 0.87; gustatory α = 0.88; bodily sensations α = 0.88; sensed presence α = 0.77.

#### Test-retest

All subscale and total scores showed acceptable test-retest correlation estimates at the *p* < 0.001 level: Auditory *r* = 0.72; Visual: *r* = 0.72; Olfactory: *r* = 0.57; Gustatory: *r* = 0.56; Bodily Sensations: *r* = 0.70; Sensed Presence: *r* = 0.69; MUSEQ Total: *r* = 0.77. In the test-retest sample, the Cronbach's alpha coefficients were estimated at: auditory α = 0.86; visual α = 0.91; olfactory α = 0.92; gustatory α = 0.93; bodily sensations α = 0.88; sensed presence α = 0.85. These results suggested stability of internal consistency over time.

### Validity

#### Convergent and divergent validity

The MUSEQ total score and modality subscale scores demonstrated good convergent validity through significant positive correlations with other measures of USE (see Table 7). Good discriminant validity was also observed via small or non-significant correlations of the MUSEQ total and subscale scores with O-LIFE-S subscales that relate to aspects of schizotypy that are not USE (i.e., CD and IA).

**Table 7:** Bootstrapped pearson correlations between MUSEQ scores and the LSHS-M-II, CAPS, and O-LIFE-S.

	**MUSEQ (*****N*** = **513)**						
	**A**	**V**	**O**	**G**	**BS**	**SP**	**Total**
LSHS-M-II (*N* = 500)	0.65^**^	0.71^**^	0.55^**^	0.51^**^	0.66^**^	0.60^**^	0.75^**^
CAPS (*N* = 498)	0.54^**^	0.61^**^	0.56^**^	0.51^**^	0.66^**^	0.50^**^	0.69^**^
O-LIFE (*N* = 503)							
UE	0.54^**^	0.60^**^	0.55^**^	0.48^**^	0.61^**^	0.63^**^	0.67^**^
CD	0.44^**^	0.36^**^	0.28^**^	0.36^**^	0.37^**^	0.22^**^	0.43^**^
IA	0.10^*^	0.08ns	0.02 ns	0.08 ns	0.05 ns	0.04 ns	0.08 ns

*MUSEQ: Multi-Modality Unusual Sensory Experiences Questionnaire, Multimodal Unusual Sensory Experiences Questionnaire; LSHS-M-II, Launay Slade Hallucination Scale-Modified-II; CAPS, Cardiff Anomalous Perception Scale; O-LIFE-S, Oxford-Liverpool Inventory of Feelings and Experiences-Short Version; UE, Unusual Experiences; CD, Cognitive Disorganization; IA, Introvertive Anhedonia*.

** p < 0.01;

* *p < 0.05; ns, not significant*.

#### Non-clinical vs. clinical group

Table 6 shows the descriptive statistics of the clinical sample. The clinical sample had significantly higher MUSEQ total scores than the non-clinical sample (*t*[543] = 5.25, *p* < 0.001, Cohen's *d* [95% CI] = 0.96 [0.59, 1.32]). The clinical sample also had significantly higher mean scores on every modality subscale compared to the non-clinical sample, with moderate to strong effect sizes (auditory: *t*[543] = 5.63, *p* < 0.001, *d* [95% CI] = 1.02 [0.66, 1.39]; visual: *t*[543] = 4.46, *p* < 0.001, *d* [95% CI] = 0.81 [0.45, 1.17]; olfactory: *t*[543] = 3.57, *p* < 0.001, *d* [95% CI] = 0.65 [0.29, 1.01]; gustatory: *t*[543] = 2.69, *p* < 0.05, *d* [95% CI] = 0.49 [0.13, 0.85]; bodily sensations: *t*[543] = 4.08, *p* < 0.01, *d* [95% CI] = 0.74 [0.38, 1.10]; and sensed presence: *t*[543] = 6.14, *p* < 0.001, *d* [95% CI] = 1.12 [0.75, 1.48]).

#### Incremental validity

Participants with sleep disorder reported: sleep apnoea (*n* = 18), insomnia (*n* = 18), night terrors (*n* = 2), sleep paralysis disorder (*n* = 1), and unspecified (*n* = 14). Those with a history of eye problems/disease (other than requiring glasses or contacts) reported the following: cataracts (*n* = 2), conjunctivitis (*n* = 2), blepharitis (*n* = 2), astigmatism (*n* = 3), floaters (*n* = 4), intraocular pressure (*n* = 2), macular degeneration (*n* = 2), amblyopia (*n* = 2), and other (e.g., cornea transplant, vitreous detachment, optic neuritis; *n* = 28). Thirty-two participants reported experiencing synaesthesia.

Table 8 shows the results of the *t*-tests between these groups and participants without a history of these variables. Participants who reported a history of sleep disorder scored significantly higher on the olfactory and gustatory subscales compared to those who did not, but their scores on other subscales and total score did not significantly differ. Individuals with a history of eye problems/disease showed significant elevations on the visual and sensed presence subscales, with non-significant differences on other modalities or total scores. The synaesthesia group scored significantly higher on all modality subscales and MUSEQ total scores compared to those who did not report experiencing synaesthesia. The ability for the MUSEQ subscales to yield group differences on different modality subscales supports its novelty in contrast to existing scales, which are designed to yield a single overall score (i.e., equivalent to the MUSEQ total score). The MUSEQ therefore appears to possess good incremental validity.

**Table 8:** Mean Differences on MUSEQ subscales and total scores in participants with and without a history of sleep disorder, eye problems/disease, and synaesthesia.

	**Auditory**	**Visual**	**Olfactory**	**Gustatory**	**Bodily sensations**	**Sensed presence**	**MUSEQ Total**
**SLEEP DISORDER**							
Yes (*N* = 53) *M (SD)*	12.19 (6.36)	11.23 (6.50)	9.58 (7.16)	8.47 (6.92)	12.08 (7.52)	4.26 (3.77)	57.81 (31.56)
No (*N* = 457) *M (SD)*	12.35 (6.13)	9.82 (7.28)	6.73 (6.0)	6.43 (5.83)	10.45 (7.52)	3.82 (3.41)	49.60 (29.76)
*t-*value	*t*(508) = 0.18	*t*(508) = 1.35	***t*****(508)** = 3.21^**^	***t*****(508)** = 2.36^*^	*t*(508) = 1.49	*t*(508) =.90	*t*(508) = 1.89
Cohen's *d* [95% CI]	0.03 [−0.31, 0.26]	0.20 [−0.09, 0.48]	0.47 [0.18, 0.75]	0.34 [0.06, 0.63]	0.22 [−0.07, 0.50]	0.13 [−0.16, 0.41]	0.27 [−0.01, 0.56]
**EYE PROBLEMS/DISEASE**							
Yes (*N* = 47) *M (SD)*	13.57 (5.10)	12.34 (7.39)	8.45 (6.48)	6.45 (6.21)	11.38 (6.81)	5.19 (4.11)	57.38 (28.12)
No (*N* = 466) *M (SD)*	12.23 (6.22)	9.77 (7.16)	6.88 (6.12)	6.66 (5.95)	10.53 (7.59)	3.73 (3.34)	49.80 (30.06)
*t-*value	*t*(511) = 1.44	***t*****(511)** = 2.34^*^	*t*(511) = 1.66	*t*(511) = 0.23	*t*(511) = 0.74	***t*****(511)** = 2.79^**^	*t*(511) = 1.66
Cohen's *d* [95% CI]	0.22 [−0.08, 0.52]	0.36 [0.06, 0.66]	0.26 [−0.05, 0.56]	0.04 [−0.34, 0.26]	0.11 [−0.19, 0.41]	0.43 [0.13, 0.73]	0.25 [−0.05, 0.55]
**SYNAESTHESIA**							
Yes (*N* = 32) *M (SD)*	15.13 (6.04)	17.13 (7.58)	11.25 (7.40)	9.97 (6.66)	17.0 (7.91)	5.03 (3.22)	75.50 (31.98)
No (*N* = 481) *M (SD)*	12.17 (6.10)	9.53 (6.94)	6.75 (5.98)	6.42 (5.86)	10.19 (7.31)	3.79 (3.44)	48.84 (29.08)
*t-*value	***t*****(511)** = 2.66^**^	***t*****(511)** = 5.96^**^	***t*****(511)** = 4.06^**^	***t*****(511)** = 3.29^**^	***t*****(511)** = 5.08^**^	***t*****(511)** = 1.99*	***t*****(511)** = 4.99^**^
Cohen's *d* [95% CI]	0.49 [0.13, 0.84]	1.09 [0.72, 1.45]	0.74 [0.38, 1.10]	0.60 [0.24, 0.96]	0.93 [0.56, 1.29]	0.36 [0.00, 0.72]	0.91 [0.55, 1.27]

Significant group differences highlighted in bold;

** p < 0.01;

* *p < 0.05; 95% CI = 95% confidence intervals*.

### Replication sample

Descriptive statistics for the replication sample yielded similar results to the initial sample (see Supplementary Table 1). The four confirmatory factor analysis models (Figure 1) were retested in the replication sample. As can be seen in Table 3, the pattern of model absolute and incremental close-fit fit indices yielded by the analyses were identical to the first study. Specifically, Models B to D yielded acceptable model fit, while Model A showed poor model fit. The correlated-factors model (Model D) showed a practical improvement in fit compared to the second-order model (Model B), but not from the bifactor model (Model C). Models B and C did not differ at the practical level (TLI difference <0.10) of improvement in model fit. Again, Models C and D fit the data well, but Model D was the most parsimonious model. Supplementary Tables 2, 3 show the factor loadings of the four models. Loadings in all four models were significant at the *p* < 0.001 level, with BS1 and O8 in Model C significant at *p* < 0.05. In Model D, the correlations between the sensory modality factors ranged from 0.56 to 0.86, with the strongest correlations yielded between the auditory and visual factors (*r* = 0.86), and the olfactory and gustatory factors (*r* = 0.86).

The omega coefficients were again estimated for Model C. Modality subscales were associated with the following ω_s_ estimates: auditory ω_s_ = 0.23 (typical); visual ω_s_ = 0.19 (relatively small); olfactory ω_s_ = 0.18 (relatively small); gustatory ω_s_ = 0.22 (typical); bodily sensations ω_s_ = 0.15 (relatively small); and sensed presence ω_s_ = 0.43 (relatively large). The ω_h_ coefficient was 0.93, confirming that in comparison to the modality subscales, the general factor again accounted for most of the reliable variance in the MUSEQ items.

## Discussion

The current study aimed to develop a reliable and valid scale that separately assesses USE in six different modalities (auditory, visual, olfactory, gustatory, bodily sensations, and sensed presence). Furthermore, it aimed to evaluate the factorial structure of these experiences and explore whether USE in different modalities could be measured separately in addition to loading onto a general factor. The development of the MUSEQ was theory-driven, and it was hypothesized that the items and modalities would be strongly related (i.e., presence of a general factor), but still uniquely accounting for some unique variance.

Confirmatory factor analyses were used to assess the psychometric quality of the MUSEQ and explore whether USE in different modalities were statistically separable. Four different models were tested: a one-factor model, second-order model, bifactor model, and correlated-factors model. In both the original sample and replication sample, the correlated-factors model and bifactor model showed similar results and yielded the best model fit. The second-order model also fit the data relatively well, but was significantly poorer than the correlated-factors model (but not the bifactor model). The one-factor model fitted the data poorly, suggesting that capturing all of the USE in the MUSEQ under one construct (i.e., a general factor) was not appropriate. All modality factors were positively correlated, which is consistent with previous findings (Launay and Slade, 1981; Morrison et al., 2000; Larøi and Van der Linden, 2005; Bell et al., 2006; Lewandowski et al., 2009; Preti et al., 2014).

All models except the unidimensional model fit well in absolute terms and yielded similar fit indices. This was expected given that the correlated-factors, second-order, and bifactor models frequently yield overlapping fit indices despite their specification of different relationships between the items (see Morgan et al., 2015). Morgan et al. (2015) suggest that approximate fit indices are useful, but that conceptual and substantive grounds must be used in determining which model most appropriately fits the data. In the current study, the bifactor model may be the preferred model compared to the correlated factors model, as the latter does not include a general factor and attributes all the variance to the modality factors. The presence of a general factor, in addition to specific modality factors, is more congruent with the current literature on USE. For example, theoretical models of visual and auditory hallucinations propose a role for both specific sensory activation and general cognitive mechanisms (see Collerton et al., 2005; Barnes and Boubert, 2008; Waters et al., 2012; Shine et al., 2014; Linszen et al., 2016).

### Modality subscales and group differences

Relating to this latter point is the issue of whether the modality factors are viable as subscales, as the presence of multidimensionality does not necessarily warrant the creation of subscores (Reise, 2012). The omega subscale coefficients (ω_s_) obtained from the bifactor model can be considered useful indicators of a factor's unique strength, independent from the general factor, and can also help determine the plausibility of subscales. In the current study, the ω_s_ values obtained in both the original sample and replication sample were relatively low to typical in range (Gignac and Kretzschmar, 2017), but do overlap with coefficients yielded by other psychological measures with strong validity (see Hull et al., 2010; Tiffin and Rolling, 2012; Dombrowski et al., 2015). Despite the dominance of a general factor and low to typical ω_s_ values, we argue that from a theoretical and conceptual point of view, the modality subscales in the MUSEQ still serve a unique function, as indicated by theoretically meaningful group differences yielded on the subscales.

For example, the current results showed individual differences in the profile of USE supporting the notion of the independence of modality factors. The community subgroups reporting the presence of eye disease, sleep disorder, and synaesthesia, each yielded a unique symptom profile. Those with eye problems or disease scored significantly higher on the visual and sensed presence subscales (and not on the other subscales), consistent with visuoperceptual deficits in this group (Schultz and Melzack, 1991; Scott et al., 2001; Collerton et al., 2005; Schwartzman et al., 2008; Vukicevic and Fitzmaurice, 2008), and findings of frequent positive correlations between visual experiences and sensed presence phenomena (Cheyne and Girard, 2007; Fénelon et al., 2011). The group with sleep disorder scored higher on the olfactory and gustatory subscales, which is in line with the reported side effects of sleep medications such as zopiclone (Ohayon, 2000). Finally, individuals with synaesthesia scored significantly higher on all modality subscales (especially visual) consistent with a blurring of sensory function (Baron-Cohen and Harrison, 1997) with a predominance of visual experiences (Novich et al., 2011; Niccolai et al., 2012).

It is important to note that significant differences in subscale scores, but not in the total score, suggests that simply calculating a total score (as is the case with current measures of USE) would fail to identify these individual differences. Thus, modality subscale scores serve a unique purpose by unmasking the presence and frequency of these experiences. The ability to measure USE in this way may be of great value in future research examining individual and group differences regarding modalities. Taking all of our findings into consideration, our hypothesis that USE may form one large constellation with a common core, but with the different modalities accounting for some unique variance, is supported.

### What does the general factor represent?

It is also important to acknowledge the dominant general factor in our model. This general factor may represent what is commonly referred to as “hallucination proneness,” which may comprise a range of factors found in individuals with higher levels of USE (e.g., schizotypal traits, sensory disturbances, cognitive difficulties, low mood, and other sociodemographic variables). It could also potentially reflect an accumulation of vulnerability risk factors, based on research suggesting that the presence of hallucinations in one modality increases the risk of hallucinations and USE in other modalities (e.g., Lim et al., 2016; Clark et al., 2017). However, further investigation is required to determine what specifically accounts for the shared variance between modalities.

### Psychometric properties of the MUSEQ

Regarding the MUSEQ more generally, results indicated good internal reliability and acceptable test-retest reliability. The test-retest results may be conservative, given that such analyses assumes a person's true score remains unchanged (Vaz et al., 2013). Participants may have experienced additional experiences on the MUSEQ in the time between measurements. Both MUSEQ total and subscale scores yielded significant positive correlations with other measures of USE indicating good convergent validity. Furthermore, weak and non-significant correlations with the CD and IA subscales of the O-LIFE-S were expected, and indicate good discriminant validity. Overall, these results indicate that the MUSEQ appears to be a reliable and valid instrument.

The MUSEQ also demonstrated good ability to discriminate between clinical and non-clinical groups. The clinical sample had significantly higher MUSEQ scores (all modalities and total) than the non-clinical sample, but with a degree of overlap in the frequency distributions. This finding supports the notion of a psychosis continuum (Johns and Van Os, 2001), and suggests that the MUSEQ may have some applicability in clinical populations. It is important to acknowledge that the subjective experience appears to differ in a qualitative sense between clinical and non-clinical samples (Stanghellini et al., 2012). Given the subjective nature of USE, however, this limitation seems applicable to all individuals, regardless of their level of pathology.

### Sensory modality findings

Other results obtained from the MUSEQ yielded some interesting findings. At the modality level, auditory and visual experiences were found to be the most common, followed by bodily sensations. Unusual auditory experiences were slightly more common than visual experiences. This outcome appears to contrast with reports that visual experiences may be more common than auditory experiences in the general population (Tien, 1991; Ohayon, 2000; McGrath et al., 2015; Peters et al., 2016). However, there are key differences between the current study and previous studies including the constructs being assessed and methodology. Specifically, the MUSEQ encompasses a continuum of USE, which contrasts with the standard screening questions in prevalence studies that are more reminiscent of clinical experiences (e.g., a single question asking about hearing sounds/voices, or seeing things that others do not). Future research may therefore be required to explore meaningful comparisons of the frequency rates of USE in different modalities.

Frequency data indicated that USE resembling “hallucination” (i.e., items at the end of each modality subscale) occurred on a monthly to annual basis in approximately 5% (for olfactory), 7% (gustatory), 17% (sensed presence), 27% (visual), and 33% (auditory and bodily sensations) of the non-clinical sample. These results are congruent with the lifetime prevalence rates of USE (i.e., hallucinations) in previous large-scale non-clinical studies (Tien, 1991; Ohayon, 2000; Nuevo et al., 2010; McGrath et al., 2015; Peters et al., 2016) and support suggestions that they do not necessarily indicate the presence of psychosis (Linscott and Van Os, 2013; Waters et al., 2017).

An additional finding of interest was the relatively high endorsement of the sensed presence items. Sensed presence experiences are under-researched, with most investigations focusing on specific populations such as the bereaved (Dewi Rees, 1971; Keen et al., 2013; Castelnovo et al., 2015), temporal lobe epilepsy (Cook and Persinger, 1997), and social isolation (Suedfeld and Mocellin, 1987). Despite ongoing debate regarding whether sensed presence experiences are illusionary, hallucinatory, or delusional in nature (Castelnovo et al., 2015), the current results add to the body of evidence showing these types of USE occur in a non-negligible number of people in the general populations (Braithwaite et al., 2013; Castelnovo et al., 2015).

### Limitations, strengths, and future directions

The current study possesses some limitations, one being that the predictive validity of the MUSEQ and its subscales was not tested (i.e., the ability for the modality subscales to predict external criterion above and beyond the general factor). The use of bifactor modeling (Model C in the current study) allows such investigations. Future studies may investigate the ability for the MUSEQ subscales to predict theoretically relevant criteria, and such research is currently being pursued by the authors. It is worth noting that the current finding of theoretically meaningful group differences on the MUSEQ subscales does provide some initial support for assessing USE in different modalities.

Another limitation is that invariance testing between groups (e.g., age, students/non-students, and different countries) was not conducted due to insufficient sample size in the required subgroups. However, previous studies have found no significant differences in the frequency of experiences between students and general communities (e.g., Lincoln and Keller, 2008), or between individuals residing in countries of a similar socioeconomic standing (McGrath et al., 2015). One exception is religious beliefs, which are associated with a higher prevalence of hallucinatory phenomena (Pelletier-Baldelli et al., 2014; Steenhuis et al., 2016). Although of interest, exploring such factors was outside the scope of this study. In regard to other invariance factors (e.g., age), only 8.5% of the current sample were older than 50 years of age, and thus it is unlikely this subsample would have significantly affected the results.

While our focus was on the six most common modalities, we are aware of other, less common, modalities such as proprioceptive, kinesthetic, vestibular, temporal, sexual, pain, and cenesthetic (Blom, 2013). There also exists multimodal USE, in which the experience occurs in more than one modality simultaneously or serially (see Lim et al., 2016). There is no doubt that investigating such experiences would be of great value, but this was beyond the scope of the current study. Future adaptations of the scale may wish to consider these other modalities and more complex multimodal experiences, or include sections allowing for qualitative responses about USE in different modalities. The authors are currently working on the addition of other dimensions to the scale such as distress, intrusiveness, and impact, and testing the reliability and validity of these modifications.

A strength of this study included a novel approach which explored the underlying structure of USE in the general population by creating a scale designed to investigate whether there is any use in separating out modalities at the measurement level. Another strength was our sample sizes, allowing sufficient exploration of USE and replication of our results in a separate sample. The MUSEQ also possesses strengths, including the ability to discriminate between different groups across modalities, and the use of a more precise frequency rating scale. Existing measures of USE calculate a total score and use response formats that do not tap into the frequency of unusual experiences, or which target frequency in a non-specific way. The features of the MUSEQ compared to the LSHS-M-II, and O-LIFE are further demonstrated in Table 9. It is hoped that the current findings and the creation of the MUSEQ will aid further investigations of USE in the general population.

**Table 9:** Comparison of the MUSEQ, CAPS, LSHS-M-II and O-LIFE-S features.

**Measure**				
	**MUSEQ**	**CAPS**	**LSHS-M-II**	**O-LIFE-S**
Focus	Unusual Sensory Experiences	Perceptual Anomalies	Hallucination Proneness	Schizotypal Traits
Development population	Non-Clinical	Non-Clinical	Non-Clinical and Clinical	Non-Clinical
Creation method	EFA, CFA	PCA	PCA, Rasch Model	EFA
Items	43	32	16	43^a^
Modality subscales	✓	✕	✕	✕
Other symptom dimensions	✕	✓(D, I, F)	✕	✓(UE, CD, IA, IN)
Response scale	Likert 0–4	Binary (No, Yes) Subscales: Likert 1–5	Likert 0–4	Binary (No, Yes)
Frequency ratings	✓	✕(Not specific time measurements)	✕	✕
USE item modalities (Number of Items)	A, V, O, G, BS, SP (7, 8, 8, 8, 8, 4)	A, V, O, G, BS, SP (9, 6, 4, 3, 6, 1)	A, V, O, BS, SP (5, 3, 1, 2, 1)	A, V, O, SP (UE) (1, 2, 1, 1)
Proneness or schizotypy items	✕	✕	✓^b^	✓^b^
Other item types	✕	Time perception; General sensory flooding; Difficulty distinguishing sensations	Hypnagogic/hypnopompic item targeting three separate modalities (not multimodal)	✕

a *Only the UE, CD, and IA subscales were used in this study (33 items)*.

b *LSHS-M-II, includes intrusive thoughts and vivid daydreaming; O-LIFE-S, includes magical powers, intrusive thoughts, cognitive disorganization, anhedonia, and antisocial behavior*.

## Conclusion

In conclusion, the MUSEQ is a reliable and valid scale that measures USE in six different modalities. Results suggested that a correlated-factors model and a bifactor model fit the data similarly well, while a one dimensional model fitted poorly. While the unique modality factors accounted for less variance than the general factor, the subscales were able to detect theoretically meaningful differences in groups of individuals with a history of eye problems/disease, sleep disorder, and synaesthesia. This has important implications for the measurement of USE, such that it allows greater specificity and better characterization of experiences while moving away from the commonly used, but poorly defined, construct of “hallucination proneness.” It also indicates that ignoring experiences in commonly under-represented modalities may be slowing progress in understanding such phenomena.

## Ethics statement

This study was carried out in accordance with the recommendations of the “National Statement on Ethical Conduct in Human Research” (National Health and Medical Research Council/Australian Research Council). All participants provided gave written informed consent in accordance with the Declaration of Helsinki. The study protocol was approved the Human Research Ethics Committee of the University of Western Australia.

## Author contributions

CM was the primary developer of the scale, conducted the analyses, interpreted the initial data, and drafted the final manuscript. MM and FW made significant contributions to the conceptual development of the scale, interpretation of the data, and critically revised drafts of the manuscript. SR and DC made significant contributions to the conceptual development of the scale and critically revised drafts of the manuscript. GG provided substantial contribution in regards to data analysis, interpretation, and critically revised drafts of the manuscript. All authors have approved the final version of this manuscript and have agreed to be accountable for all aspects of the work.

### Conflict of interest statement

The authors declare that the research was conducted in the absence of any commercial or financial relationships that could be construed as a potential conflict of interest.

## Abbreviations

*Analyses:* EFA: Exploratory Factor Analysis
CFA: Confirmatory Factor Analysis
PCA: Principal Components Analysis.
*Modalities:* A: Auditory
V: Visual
O: Olfactory
G: Gustatory
BS: Bodily Sensations
SP: Sensed Presence.
*Scales/Subscales:* LSHS-M-II: Launay Slade Hallucination Scale-Modified-II
CAPS: Cardiff Anomalous Perception Scale
D: Distress
I: Intrusiveness
F: Frequency
UE: Unusual Experiences
CD: Cognitive Disorganization
IA: Introvertive Anhedonia
IN: Impulsive Non-Conformity.
