# Supplementary material for: The Structure and Measurement of Unusual Sensory Experiences in Different Modalities: The Multi-Modality Unusual Sensory Experiences Questionnaire (MUSEQ)
Source: Front Psychol. 2017 Aug 11;8:1363. doi: 10.3389/fpsyg.2017.01363 (PMC5554527; doi:10.3389/fpsyg.2017.01363)
Supplement: Supplementary file 1 [file Table1.DOCX]

Supplementary Material

**The Structure and Measurement of Unusual Sensory Experiences in Different Modalities: The Multi-Modality Unusual Sensory Experiences Questionnaire (MUSEQ)**

**Claire A. A. Mitchell^*^, Murray T. Maybery, Suzanna N. Russell-Smith, Daniel Collerton, Gilles E. Gignac, Flavie Waters**

*** Correspondence:**Claire Mitchell
[claire.mitchell@research.uwa.edu.au](mailto:claire.mitchell@research.uwa.edu.au)

Supplementary Table 1

*Descriptive Statistics of MUSEQ for the Replication Sample*

|  | | **MUSEQ** | | | | | | |
| --- | --- | --- | --- | --- | --- | --- | --- | --- |
|  | Total | | A | V | O | G | BS | SP |
| *M*  *(SD)* | 43.38 (28.69) | | 12.12 (5.97) | 9.18 (6.96) | 5.75 (5.74) | 5.89 (6.05) | 7.84 (6.41) | 2.59 (3.10) |
| Median | 38 | | 12 | 8 | 4 | 4 | 7 | 2 |
| Mode | 25 | | 11 | 0 | 0 | 0 | 0 | 0 |
| Min - Max | 0-143 | | 0-27 | 0-32 | 0-32 | 0-32 | 0-30 | 0-16 |
| ________________________________________________________________________________ | | | | | | | | |

*N* = 659

*Abbreviations:* MUSEQ, Multi-Modality Unusual Sensory Experiences Questionnaire; A, Auditory; V, Visual; O, Olfactory; G, Gustatory; BS, Bodily Sensations; SP, Sensed Presence.
