# Supplementary material for: The Structure and Measurement of Unusual Sensory Experiences in Different Modalities: The Multi-Modality Unusual Sensory Experiences Questionnaire (MUSEQ)
Source: Front Psychol. 2017 Aug 11;8:1363. doi: 10.3389/fpsyg.2017.01363 (PMC5554527; doi:10.3389/fpsyg.2017.01363)
Supplement: Supplementary file 2 [file Table2.DOCX]

Supplementary Material

**The Structure and Measurement of Unusual Sensory Experiences in Different Modalities: The Multi-Modality Unusual Sensory Experiences Questionnaire (MUSEQ)**

**Claire A. A. Mitchell^*^, Murray T. Maybery, Suzanna N. Russell-Smith, Daniel Collerton, Gilles E. Gignac, Flavie Waters**

*** Correspondence:**Claire Mitchell
[claire.mitchell@research.uwa.edu.au](mailto:claire.mitchell@research.uwa.edu.au)

Supplementary Table 2

*Standardised Factor Loadings (WLSMV) for the One-Factor and Second-Order Models in the Replication Sample (N* = 659)

|  |  | **Model A (One-Factor)** |  | **Model B (Second-Order)** | | | | | | |
| --- | --- | --- | --- | --- | --- | --- | --- | --- | --- | --- |
| Item |  | General |  | A | V | O | G | BS | SP | General |
| ***A*** |  |  |  |  |  |  |  |  |  | .87 |
| A1 |  | .61 |  | .69 |  |  |  |  |  |  |
| A2 |  | .66 |  | .76 |  |  |  |  |  |  |
| A3 |  | .55 |  | .63 |  |  |  |  |  |  |
| A4 |  | .52 |  | .59 |  |  |  |  |  |  |
| A5 |  | .52 |  | .59 |  |  |  |  |  |  |
| A6 |  | .65 |  | .73 |  |  |  |  |  |  |
| A7 |  | .73 |  | .83 |  |  |  |  |  |  |
| ***V*** |  |  |  |  |  |  |  |  |  | .90 |
| V1 |  | .69 |  |  | .75 |  |  |  |  |  |
| V2 |  | .71 |  |  | .78 |  |  |  |  |  |
| V3 |  | .62 |  |  | .67 |  |  |  |  |  |
| V4 |  | .58 |  |  | .63 |  |  |  |  |  |
| V5 |  | .76 |  |  | .82 |  |  |  |  |  |
| V6 |  | .69 |  |  | .75 |  |  |  |  |  |
| V7 |  | .74 |  |  | .80 |  |  |  |  |  |
| V8 |  | .79 |  |  | .86 |  |  |  |  |  |
| ***O*** |  |  |  |  |  |  |  |  |  | .88 |
| O1 |  | .68 |  |  |  | .75 |  |  |  |  |
| O2 |  | .73 |  |  |  | .79 |  |  |  |  |
| O3 |  | .70 |  |  |  | .77 |  |  |  |  |
| O4 |  | .81 |  |  |  | .89 |  |  |  |  |
| O5 |  | .67 |  |  |  | .74 |  |  |  |  |
| O6 |  | .79 |  |  |  | .85 |  |  |  |  |
| O7 |  | .82 |  |  |  | .88 |  |  |  |  |
| O8 |  | .71 |  |  |  | .78 |  |  |  |  |
| ***G*** |  |  |  |  |  |  |  |  |  | .88 |
| G1 |  | .74 |  |  |  |  | .81 |  |  |  |
| G2 |  | .72 |  |  |  |  | .78 |  |  |  |
| G3 |  | .71 |  |  |  |  | .77 |  |  |  |
| G4 |  | .69 |  |  |  |  | .75 |  |  |  |
| G5 |  | .75 |  |  |  |  | .82 |  |  |  |
| G6 |  | .84 |  |  |  |  | .89 |  |  |  |
| G7 |  | .86 |  |  |  |  | .91 |  |  |  |
| G8 |  | .86 |  |  |  |  | .90 |  |  |  |
| ***BS*** |  |  |  |  |  |  |  |  |  | .92 |
| BS1 |  | .75 |  |  |  |  |  | .82 |  |  |
| BS2 |  | .66 |  |  |  |  |  | .71 |  |  |
| BS3 |  | .72 |  |  |  |  |  | .78 |  |  |
| BS4 |  | .68 |  |  |  |  |  | .74 |  |  |
| BS5 |  | .67 |  |  |  |  |  | .72 |  |  |
| BS6 |  | .52 |  |  |  |  |  | .56 |  |  |
| BS7 |  | .72 |  |  |  |  |  | .77 |  |  |
| BS8 |  | .73 |  |  |  |  |  | .79 |  |  |
| ***SP*** |  |  |  |  |  |  |  |  |  | .72 |
| SP1 |  | .68 |  |  |  |  |  |  | .92 |  |
| SP2 |  | .60 |  |  |  |  |  |  | .79 |  |
| SP3 |  | .65 |  |  |  |  |  |  | .85 |  |
| SP4 |  | .47 |  |  |  |  |  |  | .63 |  |

*Note:* Factor loadings in bold not significant (*p >* .05). Abbreviations: A, Auditory; V, Visual; O, Olfactory; G, Gustatory; BS, Bodily Sensations; SP, Sensed Presence.
