# Supplementary material for: The Structure and Measurement of Unusual Sensory Experiences in Different Modalities: The Multi-Modality Unusual Sensory Experiences Questionnaire (MUSEQ)
Source: Front Psychol. 2017 Aug 11;8:1363. doi: 10.3389/fpsyg.2017.01363 (PMC5554527; doi:10.3389/fpsyg.2017.01363)
Supplement: Supplementary file 3 [file Table3.DOCX]

Supplementary Material

**The Structure and Measurement of Unusual Sensory Experiences in Different Modalities: The Multi-Modality Unusual Sensory Experiences Questionnaire (MUSEQ)**

**Claire A. A. Mitchell^*^, Murray T. Maybery, Suzanna N. Russell-Smith, Daniel Collerton, Gilles E. Gignac, Flavie Waters**

*** Correspondence:**Claire Mitchell
[claire.mitchell@research.uwa.edu.au](mailto:claire.mitchell@research.uwa.edu.au)

Supplementary Table 3

*Standardised Factor Loadings (WLSMV) for the Bifactor and Correlated-Factors Models in the Replication Sample (N* = 659)

|  | **Model C (Bifactor)** | | | | | | |  | | **Model D (Correlated-Factors)** | | | | | | |  |
| --- | --- | --- | --- | --- | --- | --- | --- | --- | --- | --- | --- | --- | --- | --- | --- | --- | --- |
| Item | General | A | V | O | G | BS | SP | |  | | A | V | O | G | BS | SP | |
| ***A*** |  |  |  |  |  |  |  | |  | |  | .86 | .71 | .67 | .81 | .66 | |
| A1 | .61 | .31 |  |  |  |  |  | |  | | .69 |  |  |  |  |  | |
| A2 | .67 | .26 |  |  |  |  |  | |  | | .76 |  |  |  |  |  | |
| A3 | .55 | .24 |  |  |  |  |  | |  | | .63 |  |  |  |  |  | |
| A4 | .49 | .49 |  |  |  |  |  | |  | | .60 |  |  |  |  |  | |
| A5 | .49 | .48 |  |  |  |  |  | |  | | .59 |  |  |  |  |  | |
| A6 | .63 | .43 |  |  |  |  |  | |  | | .73 |  |  |  |  |  | |
| A7 | .73 | .29 |  |  |  |  |  | |  | | .82 |  |  |  |  |  | |
| ***V*** |  |  |  |  |  |  |  | |  | |  |  | .75 | .75 | .82 | .69 | |
| V1 | .68 |  | .24 |  |  |  |  | |  | |  | .75 |  |  |  |  | |
| V2 | .71 |  | .21 |  |  |  |  | |  | |  | .78 |  |  |  |  | |
| V3 | .61 |  | .26 |  |  |  |  | |  | |  | .67 |  |  |  |  | |
| V4 | .54 |  | .49 |  |  |  |  | |  | |  | .63 |  |  |  |  | |
| V5 | .74 |  | .38 |  |  |  |  | |  | |  | .82 |  |  |  |  | |
| V6 | .66 |  | .44 |  |  |  |  | |  | |  | .76 |  |  |  |  | |
| V7 | .72 |  | .36 |  |  |  |  | |  | |  | .80 |  |  |  |  | |
| V8 | .78 |  | .36 |  |  |  |  | |  | |  | .86 |  |  |  |  | |
| ***O*** |  |  |  |  |  |  |  | |  | |  |  |  | .86 | .78 | .56 | |
| O1 | .66 |  |  | .32 |  |  |  | |  | |  |  | .75 |  |  |  | |
| O2 | .70 |  |  | .41 |  |  |  | |  | |  |  | .79 |  |  |  | |
| O3 | .69 |  |  | .30 |  |  |  | |  | |  |  | .77 |  |  |  | |
| O4 | .80 |  |  | .27 |  |  |  | |  | |  |  | .88 |  |  |  | |
| O5 | .68 |  |  | .17 |  |  |  | |  | |  |  | .74 |  |  |  | |
| O6 | .70 |  |  | .60 |  |  |  | |  | |  |  | .85 |  |  |  | |
| O7 | .73 |  |  | .54 |  |  |  | |  | |  |  | .88 |  |  |  | |
| O8 | .72 |  |  | .14 |  |  |  | |  | |  |  | .78 |  |  |  | |
| ***G*** |  |  |  |  |  |  |  | |  | |  |  |  |  | .81 | .56 | |
| G1 | .72 |  |  |  | .33 |  |  | |  | |  |  |  | .81 |  |  | |
| G2 | .68 |  |  |  | .42 |  |  | |  | |  |  |  | .79 |  |  | |
| G3 | .68 |  |  |  | .33 |  |  | |  | |  |  |  | .77 |  |  | |
| G4 | .66 |  |  |  | .36 |  |  | |  | |  |  |  | .76 |  |  | |
| G5 | .72 |  |  |  | .40 |  |  | |  | |  |  |  | .82 |  |  | |
| G6 | .79 |  |  |  | .41 |  |  | |  | |  |  |  | .89 |  |  | |
| G7 | .80 |  |  |  | .44 |  |  | |  | |  |  |  | .91 |  |  | |
| G8 | .78 |  |  |  | .49 |  |  | |  | |  |  |  | .90 |  |  | |
| ***BS*** |  |  |  |  |  |  |  | |  | |  |  |  |  |  | .70 | |
| BS1 | .77 |  |  |  |  | .09 |  | |  | |  |  |  |  | .82 |  | |
| BS2 | .66 |  |  |  |  | .24 |  | |  | |  |  |  |  | .71 |  | |
| BS3 | .72 |  |  |  |  | .29 |  | |  | |  |  |  |  | .78 |  | |
| BS4 | .69 |  |  |  |  | .21 |  | |  | |  |  |  |  | .74 |  | |
| BS5 | .66 |  |  |  |  | .31 |  | |  | |  |  |  |  | .72 |  | |
| BS6 | .51 |  |  |  |  | .35 |  | |  | |  |  |  |  | .56 |  | |
| BS7 | .69 |  |  |  |  | .55 |  | |  | |  |  |  |  | .77 |  | |
| BS8 | .72 |  |  |  |  | .35 |  | |  | |  |  |  |  | .79 |  | |
| ***SP*** |  |  |  |  |  |  |  | |  | |  |  |  |  |  |  | |
| SP1 | .69 |  |  |  |  |  | .30 | |  | |  |  |  |  |  | .92 | |
| SP2 | .53 |  |  |  |  |  | .74 | |  | |  |  |  |  |  | .79 | |
| SP3 | .59 |  |  |  |  |  | .66 | |  | |  |  |  |  |  | .85 | |
| SP4 | .45 |  |  |  |  |  | .49 | |  | |  |  |  |  |  | .63 | |

*Note:* Factor loadings in bold not significant (*p >* .05). Abbreviations: A, Auditory; V, Visual; O, Olfactory; G, Gustatory; BS, Bodily Sensations; SP, Sensed Presence.
