# Supplementary material for: The Structure and Measurement of Unusual Sensory Experiences in Different Modalities: The Multi-Modality Unusual Sensory Experiences Questionnaire (MUSEQ)
Source: Front Psychol. 2017 Aug 11;8:1363. doi: 10.3389/fpsyg.2017.01363 (PMC5554527; doi:10.3389/fpsyg.2017.01363)
Supplement: Supplementary file 4 [file Table4.pdf]

Supplementary Material

The Structure and Measurement of Unusual Sensory Experiences in Different Modalities: The Multi-Modality Unusual Sensory Experiences Questionnaire (MUSEQ)

Claire A. A. Mitchell*, Murray T. Maybery, Suzanna N. Russell-Smith, Daniel Collerton, Gilles E. Gignac, Flavie Waters

*** Correspondence:**
Claire Mitchell
claire.mitchell@research.uwa.edu.au

Supplementary Table 4

Multi-Modality Unusual Sensory Experiences Questionnaire (MUSEQ)

We now know that both healthy people and people with medical or psychological conditions report having strange or unusual experiences. These experiences range from being very subtle to quite obvious. We want to find out more. Please answer **all** questions by **placing a tick or cross (✓ or X)** in the most appropriate response box. There are no right/wrong answers and your responses are kept confidential.

**AUDITORY**					
**There have been times when…**	***Never*** (Never happened)	***Hardly Ever*** (Once or twice in my life**)**	***Rarely*** (Once or twice a year)	***Occasionally*** (A few times a year)	***Frequently*** (At least monthly)
**1.** My ears have played tricks on me					
**2.** Sounds were louder than they normally would be					
**3.** I thought of a song and could almost hear it with distinct clarity					
**4.** I was in a crowd or with other people and heard my name being called, only to find that I was mistaken					
**5.** I have heard my phone ring then found that it wasn't ringing at all					
**6.** I could hear sounds, music, or noises that other people could not hear					
**7.** I have heard a person's voice and then found that no-one was there					
**VISUAL**					
**There have been times when…**	***Never*** (Never happened)	***Hardly Ever*** (Once or twice in my life)	***Rarely*** (Once or twice a year)	***Occasionally*** (A few times a year)	***Frequently*** (At least monthly)
**8.** My eyes have played tricks on me					
**9.** I found that lights or colours seem brighter or more intense than they normally would be					
**10**. I thought of people, objects, or landscapes, and could almost see their image in front of my eyes					
**11.** I have looked at a patterned object (e.g., wallpaper, curtains,					

tiled floor) and a figure or face has emerged					
**12.** I have seen lights, flashes, or other shapes that other people could not see					
**13.** I looked at an object and it transformed itself before my eyes into something else					
**14.** I saw a brief image of an object, animal, or person pass me by in my peripheral vision, but when I looked there was nothing there					
**15.** I saw people, faces, or animals, and then found that nothing was there					
**OLFACTORY**					
**There have been times when…**	***Never*** (Never happened)	***Hardly Ever*** (Once or twice in my life)	***Rarely*** (Once or twice a year)	***Occasionally*** (A few times a year)	***Frequently*** (At least monthly)
**16.** My nose (sense of smell) has played tricks on me					
**17.** I thought that everyday smells were unusually strong					
**18.** I thought of a smell and I could almost smell it for real					
**19.** Common smells seemed unusually different					
**20.** I noticed the smell of smoke, burning, or gas when there was nothing there					
**21.** I have suddenly been struck by an unpleasant or disgusting smell that no-one else could smell					
**22.** I have suddenly been struck by a very pleasant smell that no-one else could smell					
**23.** I have been struck with the smell of odd things which I interpreted as death, colours, or ghosts					
**GUSTATORY**					
**There have been times when…**	***Never*** (Never happened)	***Hardly Ever*** (Once or twice in my life)	***Rarely*** (Once or twice a year)	***Occasionally*** (A few times a year)	***Frequently*** (At least monthly)

**24**. My sense of taste has played tricks on me					
**25.** I thought that food or drink tasted stronger than it normally would					
**26.** I thought of a taste and found that I could taste it in my mouth as if it was real					
**27.** I ate the same food as another person and thought it tasted off, but the other person did not seem to think so					
**28.** I have consumed food or drink and it tasted like something completely different					
**29.** I had nothing in my mouth but I suddenly tasted something very confusing which faded very quickly					
**30.** I had nothing in my mouth but I suddenly tasted something unpleasant which was really persistent					
**31.** I had nothing in my mouth but I suddenly tasted something very pleasant which was really persistent					
**BODILY SENSATIONS**					
**There have been times when…**	***Never*** (Never happened)	***Hardly Ever*** (Once or twice in my life)	***Rarely*** (Once or twice a year)	***Occasionally*** (A few times a year)	***Frequently*** (At least monthly)
**32.** My body senses have played tricks on me					
**33.** I found my skin to be more sensitive to cold, heat, or touch than usual					
**34.** I thought of a touch or other sensations on my skin and almost felt it on my skin					
**35.** I have experienced the sensation that my body (or part of my body) was different in shape or size					
**36.** I could feel burning, tingling, scraping, or heat on my skin, although there was nothing causing it					

**37.** I have felt things moving or crawling on or under my skin					
**38.** I have experienced the sensation that something was pressing on my skin, or that I was holding an object in my hand, but then found there was nothing there					
**39.** I have felt someone or something touching me, but when I turned to look there was nothing there					
**SENSED PRESENCE**					
**There have been times when…**	***Never*** (Never happened)	***Hardly Ever*** (Once or twice in my life)	***Rarely*** (Once or twice a year)	***Occasionally*** (A few times a year)	***Frequently*** (At least monthly)
**40.** I felt the presence of someone, even though I could not see them (e.g., behind me, or in another room)					
**41.** I have felt an unseen evil presence around me					
**42.** I have felt an unseen angelic presence around me					
**43.** I have felt the presence of a relative or friend who has passed away					

Thank you for your participation, it is greatly appreciated.

<u>MUSEQ Scoring Procedure</u>

The MUSEQ contains 43-items, which fall into six modality subscales. Both subscale total scores and a MUSEQ total score can be calculated.

- Subscale scores are obtained by summing item responses for the respective subscale.
- Total scores are obtained by summing all subscale scores.

Subscale and scoring information is as follows:

AUDITORY
Items: 7
Minimum Score: 0
Maximum Score: 28

VISUAL
Items: 8
Minimum Score: 0
Maximum Score: 32

OLFACTORY
Items: 8
Minimum Score: 0
Maximum Score: 32

GUSTATORY
Items: 8
Minimum Score: 0
Maximum Score: 32

BODILY SENSATIONS
Items: 8
Minimum Score: 0
Maximum Score: 32

SENSED PRESENCE
Items: 4
Minimum Score: 0
Maximum Score: 16

<u>MUSEQ TOTAL</u>
Items: 43
Minimum Score: 0
Maximum Score: 172
